# Culture Medium and Sex Drive Epigenetic Reprogramming in Preimplantation Bovine Embryos

**DOI:** 10.3390/ijms22126426

**Published:** 2021-06-15

**Authors:** Sebastian Canovas, Elena Ivanova, Meriem Hamdi, Fernando Perez-Sanz, Dimitrios Rizos, Gavin Kelsey, Pilar Coy

**Affiliations:** 1 Physiology of Reproduction Group, Physiology Department, Mare Nostrum Campus, University of Murcia, 30100 Murcia, Spain; scber@um.es; 2 Biomedical Research Institute of Murcia, IMIB-Arrixaca-UMU, 30120 Murcia, Spain; fernando.perez8@um.es; 3 Epigenetics Programme, The Babraham Institute, Cambridge CB22 3AT, UK; elena.ivanova@babraham.ac.uk (E.I.); gavin.kelsey@babraham.ac.uk (G.K.); 4 Animal Reproduction Department, National Institute for Agriculture and Food Research and Technology, INIA, 28040 Madrid, Spain; mhamdi9186@hotmail.com (M.H.); drizos@inia.es (D.R.)

**Keywords:** embryo, DNA methylation, epigenetic, preimplantation, sexual dimorphism, bovine

## Abstract

Assisted reproductive technologies impact transcriptome and epigenome of embryos and can result in long-term phenotypic consequences. Whole-genome DNA methylation profiles from individual bovine blastocysts in vivo- and in vitro-derived (using three sources of protein: reproductive fluids, blood serum and bovine serum albumin) were generated. The impact of in vitro culture on DNA methylation was analyzed, and sex-specific methylation differences at blastocyst stage were uncovered. In vivo embryos showed the highest levels of methylation (29.5%), close to those produced in vitro with serum, whilst embryos produced in vitro with reproductive fluids or albumin showed less global methylation (25–25.4%). During repetitive element analysis, the serum group was the most affected. DNA methylation differences between in vivo and in vitro groups were more frequent in the first intron than in CpGi in promoters. Moreover, hierarchical cluster analysis showed that sex produced a stronger bias in the results than embryo origin. For each group, distance between male and female embryos varied, with in vivo blastocyst showing a lesser distance. Between the sexually dimorphic methylated tiles, which were biased to X-chromosome, critical factors for reproduction, developmental process, cell proliferation and DNA methylation machinery were included. These results support the idea that blastocysts show sexually-dimorphic DNA methylation patterns, and the known picture about the blastocyst methylome should be reconsidered.

## 1. Introduction

Early embryo development is one of the most dynamic and complex biological processes. During this period, dramatic transcriptomic, epigenetic, physiological and morphological changes occur in a short temporal window. Indeed, early embryos show the capacity to respond to maternal environmental changes (embryonic plasticity), which is considered as an evolutionary strategy for adaptation [1]. Nonetheless, non-maternal-related changes in the environment of the developing embryos, as conditions derived from assisted reproductive technologies (ART), could result in long-term phenotypic consequences in adult life, a concept defined as “developmental programming” [2].

Since the 1980s, a variety of approaches has been tested to improve in vitro embryo production (IVP), with the outcome being measured as their capacity to establish a pregnancy. In recent years, in contrast, there has been a growing concern about the health of in vitro-derived offspring, because there is increasing evidence showing that in vitro culture can have a significant impact on the transcriptional and epigenetic profiles of the embryo. Increased risk of fetal growth restriction, premature birth, low birthweight, perinatal complications and/or congenital anomalies (including genetic and non-genetic) have been reported in humans [3,4,5], cows [6] and mice [7,8,9,10].

Amongst the strategies for improving the quality of the in vitro-derived embryos, the supplementation of culture media with different oviductal and endometrial factors has been considered. Several cytokines have shown their capacity to influence embryo programming and epigenetic marks [11]. For example, in vitro-derived embryos treated with colony-stimulation factor 2 (CSF2) reached higher blastocyst rate and higher survival after embryo transfer [12,13,14,15,16,17]. Furthermore, the culture of bovine embryos with CSF2 restores to normal the expression of genes in liver and placenta that are deregulated by embryo culture alone [18]. Thus, oviductal and endometrial factors can improve embryo quality and could act as developmental programming agents. However, taking into account the complexity of the oviductal and uterine fluids (with more than 1600 proteins identified only in the oviduct [19], it seems difficult to accept that the use of just a few factors in isolation can reverse all undesired consequences of the IVP.

In accordance with this complexity, in pigs, the use of whole reproductive fluids as additives in the culture media showed a significant increase in quality of the blastocysts produced, with gene expression and DNA methylation patterns closer to in vivo blastocysts [20]. In cattle, the use of reproductive fluids has been shown also to have some benefits on the quality of the embryos produced [21,22], although it is unknown whether the DNA methylation patterns are also better reprogrammed in embryos produced in the presence of these fluids. Therefore, one of the objectives of the present study was to investigate the effect of reproductive fluids on the epigenetic status (genome-wide DNA methylation) of IVP bovine blastocysts.

Sex-specific transcriptional differences between males and females have been described in in vivo- and in vitro-derived embryos [23,24,25]. However, few studies have analyzed epigenetic differences between male and female preimplantation embryos, and sexual dimorphism in DNA methylation is only well documented after sex determination. During the period of early embryo development, extensive DNA methylation reprogramming occurs, and the exposure to stressful conditions, under in vivo (i.e., in utero exposure to maternal undernutrition) or in vitro conditions, can produce deviations in the DNA methylome [26,27,28,29,30,31]. Considering all these facts, we investigated the sexual epigenetic dimorphism of the bovine embryos in vivo collected or in vitro produced. We wondered if a sex-specific impact of environmental conditions during in vitro culture exists or not. For both purposes, genome-wide DNA methylation was analyzed in single male and female bovine blastocysts either in vivo collected or in vitro produced using three different types of additives for the embryo culture media (reproductive fluids, fetal calf serum or bovine serum albumin). The results showed that both culture media composition and sex of the embryos affect DNA methylation patterns at as early as day 8 of development.

## 2. Results

### 2.1. Global DNA Methylation Landscape in Single Bovine Blastocysts Obtained after In Vitro Production (IVP)

**Figure 1:**
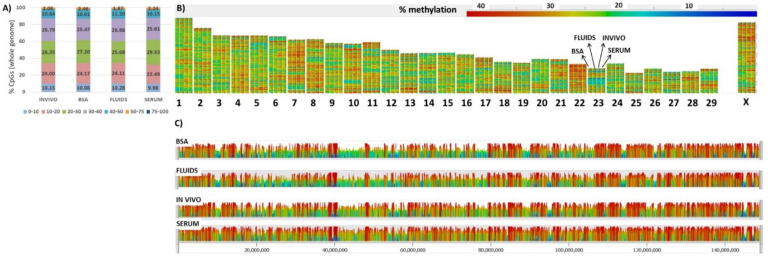
(**A**) Distribution of methylation percentages across tiles of 100 CpGs on the bovine blastocyst by group. (**B**) Domainogram shows quantitation of the blastocyst methylation over the whole genome for each group (BSA, FLUIDS, INVIVO, SERUM) at every chromosome (1–29, X). (**C**) Browser shot of methylation landscape of the four groups analyzed (whole X-chromosome).

We were interested to evaluate how various IVP protocols influenced the DNA methylation landscape of bovine blastocysts. Individual blastocysts produced by IVF and cultured with fetal calf serum (SERUM), bovine serum albumin (BSA) or reproductive fluids (FLUIDS) were collected at day 7 after IVF; in addition, in vivo grown blastocysts (INVIVO) were flushed at day 7 for a reference group (Figure 10). Whole-genome DNA methylation profiles of four individual bovine blastocysts per group were generated by PBAT; each group was ascertained to contain two male and two female blastocysts by sequence reads mapping to Y-chromosome loci. The number of unique sequence alignments obtained per sample ranged from 17,142,606 to 72,901,316, giving a coverage of CpGs (≥1 read) from 47.66% to 81.95% (Supplementary Table S1). The global CpG methylation level was 19.5–34% (Supplementary Table S2), in line with expectations (Ivanova et al., 2020). Within this range, the INVIVO group had higher methylation values (29.5 ± 2.2%) than any of the in vitro groups (SERUM: 28.1 ± 2.6%; BSA: 25.4 ± 2%; FLUIDS: 25 ± 1.6%). Methylation percentages in the CHG context ranged from 0.5 to 1.7 and in the CHH context from 0.4 to 1.5 (Supplementary Table S2), as expected, and indicated a very high bisulfite conversion rate in all libraries (>98.3%). Analysis of methylation in fixed tiles of 100 CpGs across the whole genome showed a similar distribution of methylation in the four groups (Figure 1A). Inspection of the methylation profiles of individual chromosomes also showed a similar global distribution in the four groups, although some differences were apparent, which were more evident in the X chromosome even though the number of male and female blastocysts was the same in all groups (Figure 1B,C).

### 2.2. DNA Methylation Differences in Bovine Blastocysts under In Vitro vs. In Vivo Culture

**Figure 2:**
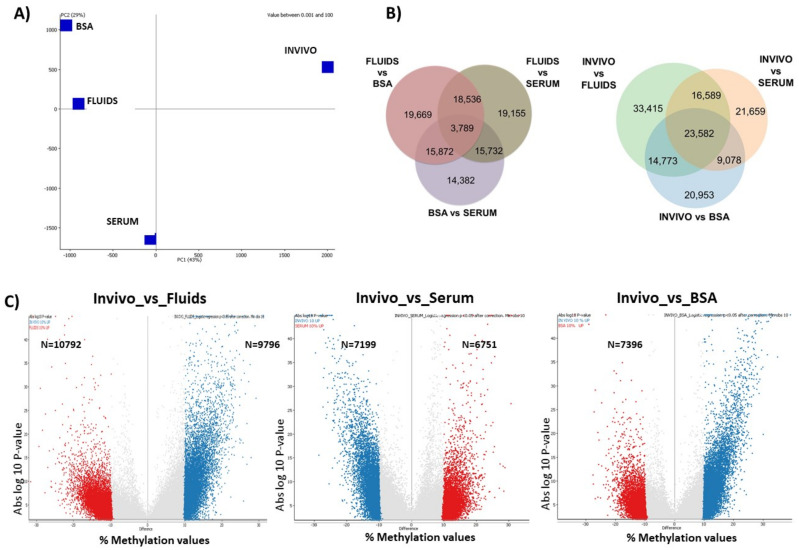
(**A**) Principal component analysis. (**B**) Venn diagrams show shared DMTs between pair-wise comparison. (**C**) Volcano plots show absolute log *p*-value vs. % methylation values differences for INVIVO blastocyst vs. FLUIDS, SERUM or BSA.

The analysis above of the global methylation patterns suggested some differences between groups. This was reinforced by principal component analysis (PCA), which separated the INVIVO from the in vitro groups on the major axis (PC1, 43% of variance), and separated the three in vitro groups on PC2 (29% of variance; Figure 2A).

Unbiased analysis of the differentially methylated tiles (DMTs) between groups revealed that after filtering DMTs with differences over 10%, which could be established as a threshold to expect some biological relevance, the SERUM group vs. INVIVO showed the lowest rate of DMTs (19.67%; Chi-square, *p* < 0.001; Table 1). Furthermore, BSA and FLUIDS groups showed a similar rate of DMTs vs. INVIVO, which was around 23% (Table 1). DMTs for each in vitro group compared to the INVIVO group are shown in Dataset 1. Pair-wise comparisons between the three in vitro groups revealed that BSA vs. SERUM showed the lowest rate of DMTs (15.13%) with methylation differences over 10%. The correlation matrix confirmed the highest similarity between some BSA and FLUIDS blastocysts (Supplementary Figure S1). Regarding the percentage of DMTs shared in the three in vitro groups with respect to INVIVO, this was 16.84% (23,582). Moreover, a percentage of 14.96% (20,953), 15.46% (21,659) and 23.86% (33,415) of all DMTs detected (140,048) were specifically differentially methylated in BSA, SERUM and FLUIDS, respectively, compared with INVIVO (Figure 2B). Volcano plots show significance of the DMTs for each in vitro group vs. INVIVO group (Figure 2C).

To better characterize the changes in methylation that resulted from the in vitro culture, overlapped subsets of DMTs (with methylation differences over 10%) were obtained by combining the three DMT lists, and it showed 2614 DMTs (Dataset 2). These DMTs were annotated to the overlapping genes and analyzed for statistical overrepresentation. Main GO biological functions overrepresented included intracellular signal transduction and regulation of cell differentiation, whereas GO molecular functions included hydrolase activity, GTPase activity, actin binding and phospholipid binding.

### 2.3. Targeted Analysis (Differentially Methylated Genomic Elements)

**Figure 3:**
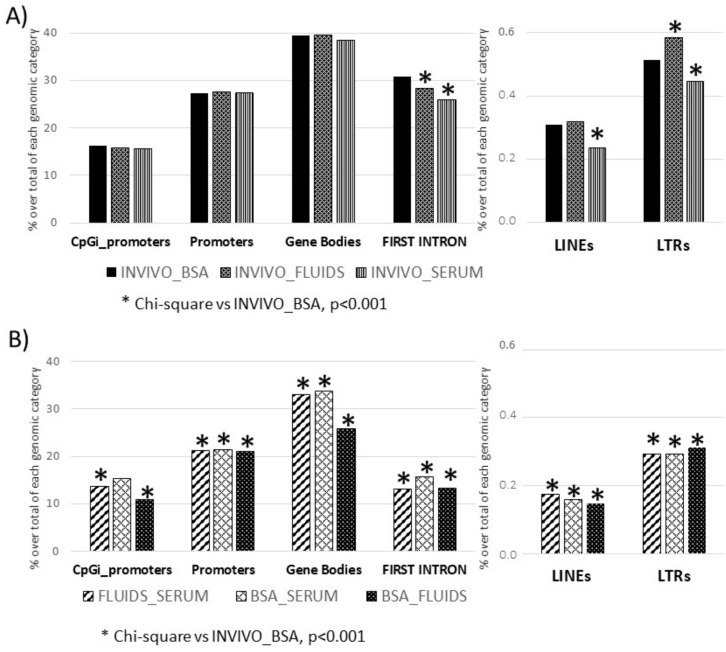
Differentially methylated genomic features after targeted analysis. (**A**) Each invitro group (BSA, FLUIDS and SERUM) was compared vs. INVIVO group. Frequency distribution of DMTs in INVIVO vs. BSA was the reference for Chi-square analysis. (**B**) Differentially methylated genomic features for pair wise comparisons between in vitro groups.

Using targeted analysis, we limited the analysis to certain loci or regions of the genome, such as promoter regions, CpGi in promoters, etc., which showed only a partial picture of the methylome but helped to identify if methylation differences were associated with some genomic elements and if they could be linked to relevant functions. This analysis showed that approximately 30% of the promoters and first introns, and 15% of the CpGi placed in promoters, were differentially methylated in the in vitro groups (BSA, SERUM and FLUIDS) vs. INVIVO group. For gene bodies, this value reached 40% over all gene bodies. In the case of the repetitive elements, the rate of differentially methylated tiles in each in vitro group (BSA, SERUM and FLUIDS) vs. INVIVO group was around 0.3% for LINEs and 0.5% for LTRs. Using as reference, the differentially methylated genomic elements between INVIVO and BSA group (conventional in vitro group), Chi-square analysis was performed. The number of differentially methylated first introns and LTRs was statistically different in FLUIDS and SERUM groups. LINEs also showed significant differences in the SERUM group (Figure 3A). Moreover, analysis of the similarities and differences between the three in vitro groups, by pair-wise comparison, revealed a lower number of differentially methylated genomic elements between in vitro groups than between INVIVO and BSA, with the exception of CpGi promoters for the comparison between BSA and SERUM (Figure 3B).

**Figure 4:**
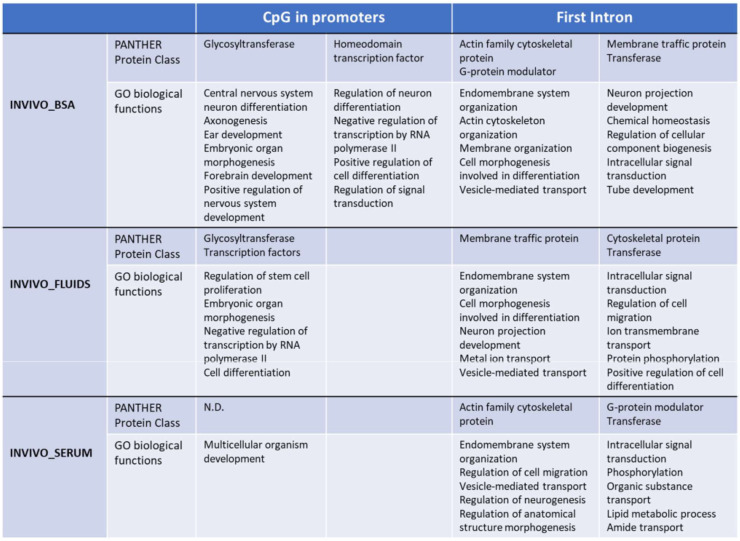
Functional annotation using PANTHER. GO biological functions and Panther pathways overrepresented in the CpG islands in promoters or FIRST introns with differential methylation between INVIVO and the other three groups are shown. Fisher’s exact test using the Bonferroni correction for multiple testing was applied for statistical overrepresentation analysis. N.D: not detected.

Functional analysis by PANTHER v.14.0 of the differentially methylated CpG islands in promoters between INVIVO vs. each in vitro group revealed enrichment in some protein classes and Gene Ontology (GO) biological processes (Figure 4). Glycosyltransferase and transcription factor protein classes were identified in the INVIVO vs. BSA and INVIVO vs. FLUIDS groups. Regarding GO biological process identified after enrichment analysis, embryonic organ morphogenesis, cell differentiation and regulation of transcription by RNA polymerase II were shared between INVIVO vs. BSA and INVIVO vs. FLUIDS comparisons. In the INVIVO vs. SERUM pair-wise, no protein classes were enriched, and the multicellular organism development biological process was the only one enriched.

Considering that a consistent correlation between DNA methylation of the first intron and gene expression across tissues and species has been reported recently [32], we also performed functional analysis of the first introns differentially methylated between the INVIVO group and the other three groups. Top ten GO biological functions are included in Figure 4. Cytoskeletal protein and transferase protein classes were overrepresented in the three pair-wise comparisons. G-protein modulator and membrane traffic protein classes were overrepresented in two pair-wise comparisons. Regarding overrepresented GO biological functions, endomembrane system organization, vesicle-mediate transport and intracellular signal transduction were present in the three pair-wise comparisons. In addition, protein phosphorylation overrepresentation was statistically significant for INVIVO_FLUIDS and INVIVO_SERUM comparisons. Other biological functions related with development, morphogenesis, ion transmembrane transport or lipid metabolic process were overrepresented in each pair-wise comparison (Figure 4).

### 2.4. DNA Methylation Differences in Female versus Male Bovine In Vivo- or In Vitro-Derived Blastocysts

**Figure 5:**
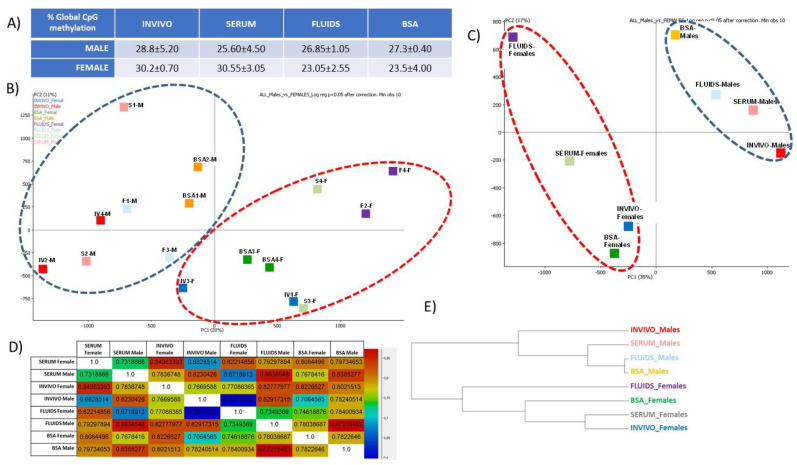
DNA methylation in female versus male bovine in vivo- or in vitro-derived blastocysts. (**A**) Global CpG methylation values for sexed blastocyst in each group. (**B**) PCA for individual blastocysts. F and M suffixes in the blastocyst ID label the sex (F: female; M: male). (**C**) PCA for samples split by culture group and sex (sub-groups). (**D**) Correlation coefficient between each subgroup. (**E**) Hierarchical clustering by subgroups.

Sexual dimorphism is a complex biological phenomenon that remains partially unclear. Although some evidence suggest that the establishment of sexually dimorphic DNA methylation patterns could occur even before the blastocyst stage, few studies have analyzed it [33,34]. Our results show that global DNA methylation values in INVIVO preimplantation blastocysts were 28.8 ± 5.20% and 30.2 ± 0.70% in male and female blastocysts, respectively. For the in vitro groups, male blastocysts in the SERUM group also displayed lower values of global CpG methylation than females. By contrast, in the FLUIDS and BSA groups, CpG methylation levels were higher in male than female blastocysts (Figure 5A). Whether these data reflect different stress levels derived from embryo culture is an option to be explored and will be addressed in the discussion.

In the PCA plot (Figure 5B), it was observed that male and female blastocysts clustered separately, irrespectively of the culture group. By combining the replicates, to minimize the individual variability, female and male blastocyst also clustered separately, and the culture group showed a lower impact than the sex for clustering (Figure 5C). Higher correlation coefficients were shown between embryos from same sex, irrespectively of the culture group. The correlation between INVIVO female vs. male blastocysts was 0.767, which was lower than the correlation for any pair-wise comparison between blastocysts from the same sex (Figure 5D).

### 2.5. Unbiased Analysis

**Figure 6:**
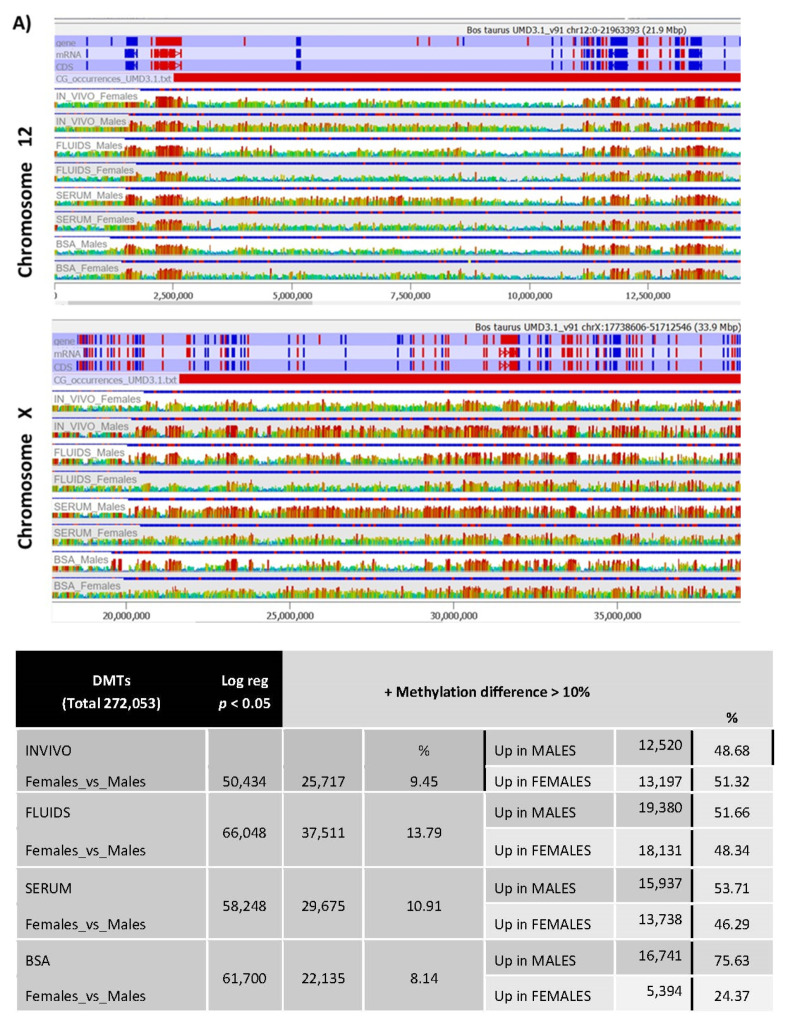

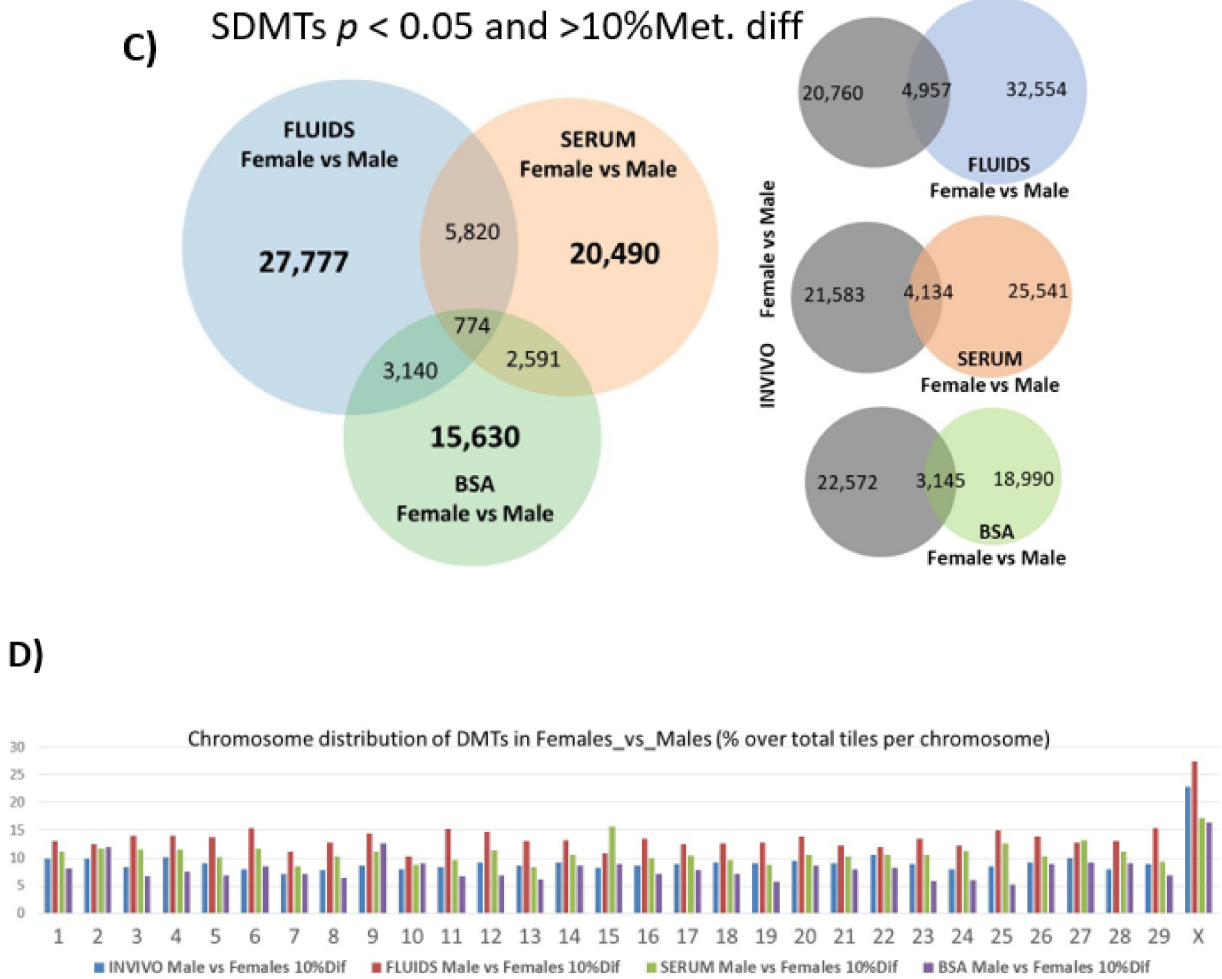

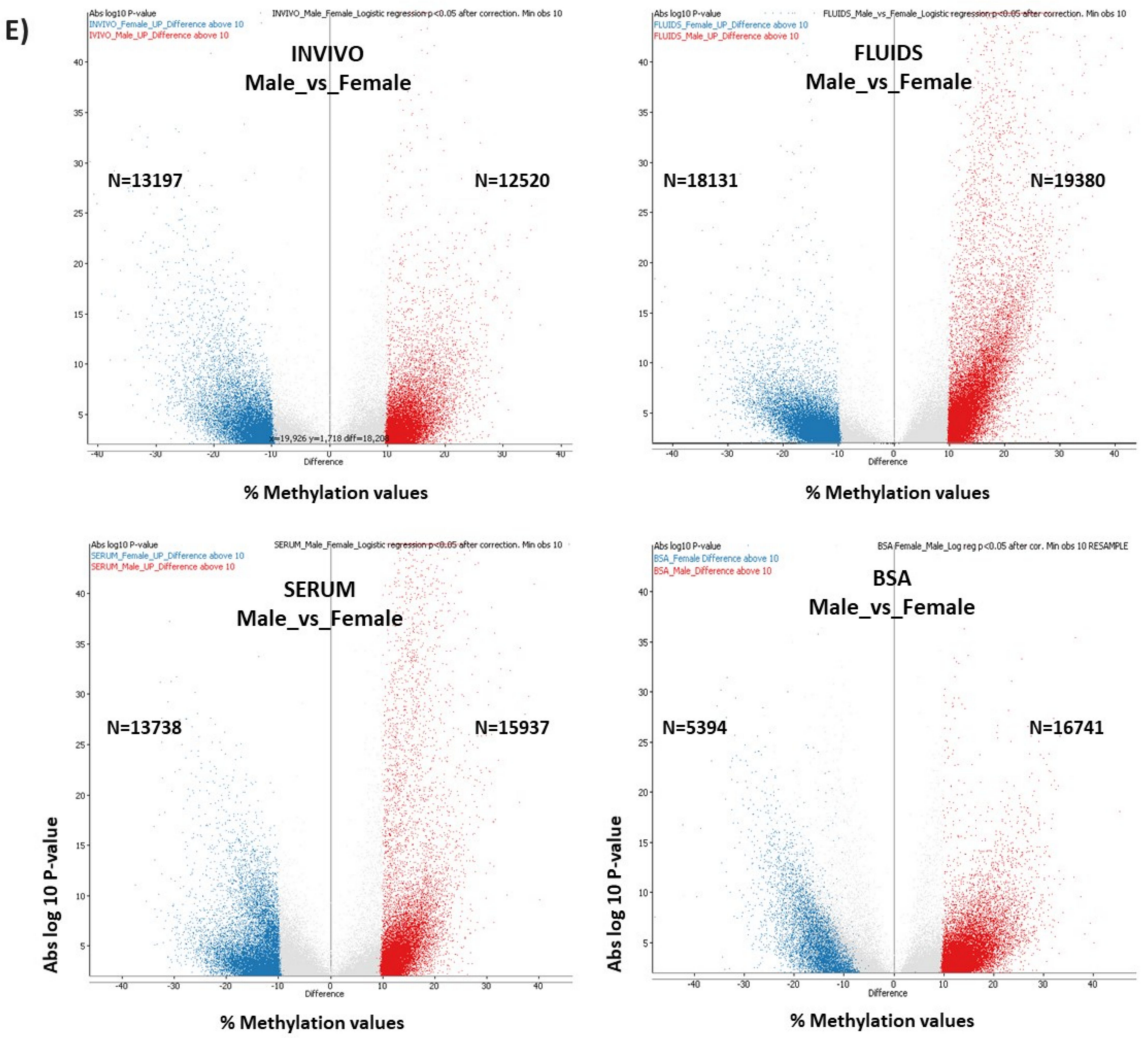
(**A**) Screenshot of Seqmonk genome browser at regions of Chromosome 12 and Chromosome X show examples of methylation differences between male and female blastocysts in each group and between groups. (**B**) DMTs of male and female blastocyts in each experimental group after logistic regression (*p* < 0.05). Indeed, these DMTs were filtered by methylation differences over 10% of methylation. (**C**) Venn diagrams show DMTs for each pair-wise comparison. (**D**) Percentage of DMTs over total tiles per chromosome in female vs. male blastocysts for each group. (**E**) Volcano Plots.

Unbiased analysis was performed to obtain a global picture of the methylation differences between male and female blastocysts for each group. A global view of the chromosomes, as shown in the domainogram (Supplementary Figure S2), allowed the detection of some differences at this level. A more detailed view by chromosome of these differences is shown for Chromosomes 12 and X (Figure 6A).

All sexually dimorphic methylated tiles (SDMTs) between male and female blastocysts inside each subgroup (INVIVO, BSA, SERUM, FLUIDS) were identified by logistic regression (Figure 6B; Dataset 3). The INVIVO blastocyst showed 50,434 sexually dimorphic methylated regions between male and female blastocysts. From these SDMTs, approximately 50% (25,717 tiles) showed methylation differences over 10%. For the three in vitro groups, the number of SDMT with methylation differences over 10% was in the same order of magnitude (from 22,135 in BSA to 37,511 in FLUIDS; Figure 6B). When SDMTs were split in tiles with higher methylation in male than in females, in all groups the percentage was around 50%, except in the BSA group with 75.63% of SDMTs with higher methylation in males. Shared SDMTs for each pair-wise comparison are shown in the Figure 6C. The three in vitro groups shared 774 SDMTs, which represent between 2% and 3.5% from all DMT with differential methylation values over 10% between male and female blastocysts. A total of 2652 tiles (10.3% of the 25,717 SDMTs) identified in the INVIVO group are located in the X chromosome, which represents more than double the number of DMTs located in any other chromosome. For all four groups, chromosomal distribution of the DMTs followed a similar tendency (Figure 6).

Volcano plots (Figure 6E) show statistically significant SDMT (*p* < 0.05 after correction and methylation values differences over 10%), illustrating higher significance (abs log 10 *p*-value) in SDMTs in females than in males, mainly in the SERUM and FLUIDS groups.

Statistically significant SDMT (*p* < 0.05 after correction and methylation values differences over 10%) for each group are shown in Dataset 3. After annotation by overlapping genes, from these SDMT, in the INVIVO group, 93 factors were related with reproduction, including 21 factors with roles in the meiotic cell cycle, and 51 involved in gamete generation (23 in male gamete). Moreover, several SDMTs reflect sexual dimorphic differences in genes involved in the DNA methylation machinery. SDMTs overlapping DNMT1, TET2, TET3 and ZFP57 showed higher methylation in females than in male INVIVO blastocysts, whilst in the case of the SDMTs overlapping TET1, those displayed higher methylation in male INVIVO blastocysts. Additionally, two SDMTs overlapping UHRF2, a factor involved in cell cycle regulation, revealed higher methylation in INVIVO females. In the FLUIDS group, SDMTs overlapping DNMT3A and UHRF2 show higher DNA methylation in female than male blastocysts, whilst TET2 and DNMT1 showed opposite results. TET2 in the SERUM group and DNMT1 in the BSA group also showed SDMT with higher methylation in male blastocysts. Contrarily, TET1 in the BSA group and TET3 in the SERUM group showed higher methylation in female blastocysts (Table 2).

In order to analyze if there is sex-dependent impact/susceptibility for specific culture conditions, we also analyzed SDMTs for each culture media pair-wise comparison, separately in female and male blastocysts (Table 3). Female blastocysts INVIVO vs. FLUIDS revealed the higher percentage of DMTs (17.63%). The percentage of tiles with higher methylation in each pair-wise comparison was equally distributed (46.17–58.59%) between the groups under comparison, for female and male blastocysts. There was an exception in the pair-wise FLUIDS vs. BSA, where around three quarters of the DMRs shower higher methylation in the FLUIDS group compared to BSA groups, in both sexes. Curiously, the BSA group was also the unique group, which showed around 75% of SDMTs with higher methylation in males, whilst in the other groups, the percentage was around 50%.

**Figure 7:**
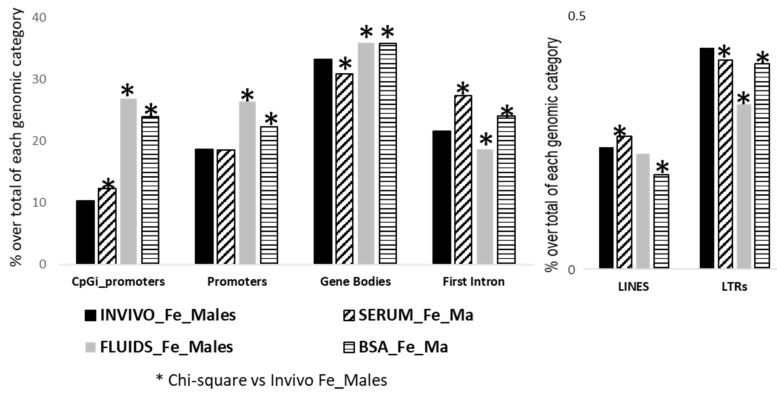
Differentially methylated genomic features after targeted analysis for male and female blastocyts in each group. Frequency distribution of DMTs in INVIVO female vs. males was the reference for Chi-square analysis, * *p* < 0.001.

**Figure 8:**
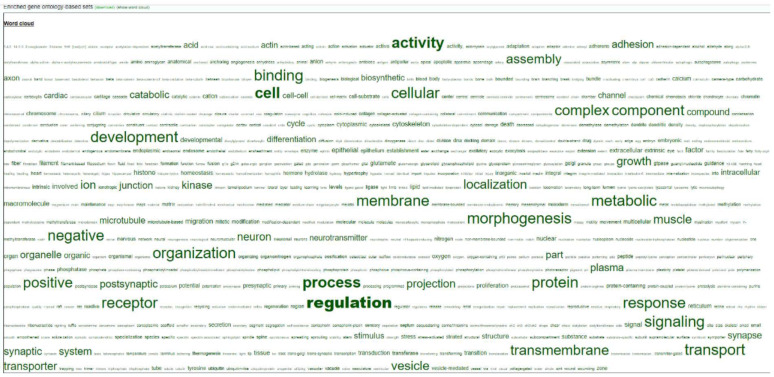
Word cloud for enriched gene ontology-base sets in female vs. male INVIVO blastocyst.

**Figure 9:**
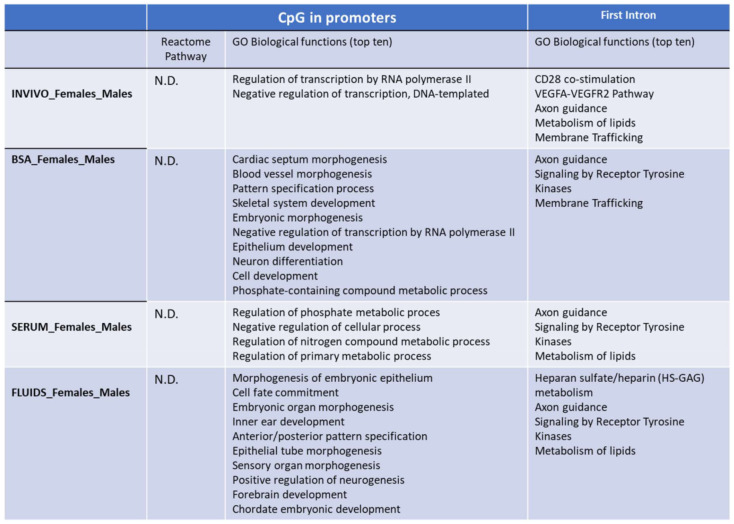
Functional annotation using PANTHER. GO biological functions and Panther pathways overrepresented in the CpG in promoters or FIRST introns with differential methylation between male and female blastocyst for each group. Fisher’s exact test using the Bonferroni correction for multiple testing was apply for statistical overrepresentation analysis. N.D: not detected.

Finally, we also performed a targeted analysis to know if SDMT are biased to specific genomic elements. The three in vitro groups showed significant differences in the number of SDMTs in all genomic features analyzed, except in promoters for the SERUM group and for LINES in the FLUIDS group (Figure 7). In order to identify if there was an overrepresentation of the SDMT between male and female blastocysts, functional annotation and statistical overrepresentation analysis were performed. A word cloud for enriched gene ontology-base sets in female vs. male INVIVO blastocyst is represented in Figure 8, which provides a useful concise visual summary. In addition, different pathways overrepresented in the SDMT were identified by PANTHER. Membrane trafficking, metabolism of lipids, axon guidance and signaling by receptor tyrosine kinase were shared by two or more groups (Figure 9).

## 3. Materials and Methods

Unless otherwise stated, all chemicals were purchased from Sigma Aldrich Quimica S.A. Company (Madrid, Spain).

### 3.1. Oocyte Collection and IVP

Immature cumulus–oocyte complexes (COCs) were obtained by aspirating follicles (diameter 2–8 mm) from ovaries of mature heifers collected at a local slaughterhouse. Groups of 50 COCs were selected and cultured in 500 μL maturation medium in four-well dishes (Nunc) for 24 h at 38.5 °C under an atmosphere of 5% CO_2_ in air, with maximum humidity. The maturation medium consisted of TCM199 supplemented with 10% (*v/v*) fetal calf serum (FCS) and 10 ng/mL epidermal growth factor.

After 24 h of maturation, in vitro fertilization (IVF) was performed as described previously [35]. Briefly, frozen semen straws from an Asturian Valley bull (ASEAVA, Asturias, Spain) previously tested for IVF, were treated with Bovipure™ (Nidacon, Sweden). Sperm concentration was determined and adjusted to a final concentration of 1 × 10^6^ spermatozoa/mL. Gametes were co-incubated for 18–20 h in 500 μL fertilization medium (Tyrode’s medium (Parrish 2014) with 25 mM bicarbonate, 22 mM sodium lactate, 1 mM sodium pyruvate and 6 mg/mL fatty acid-free BSA (Sigma A6003) supplemented with 10 mg/mL heparin sodium salt (Calbiochem) in a four-well dish in groups of 50 COCs per well under an atmosphere of 5% CO_2_ in air, with maximum humidity at 38.5 °C.

**Figure 10:**
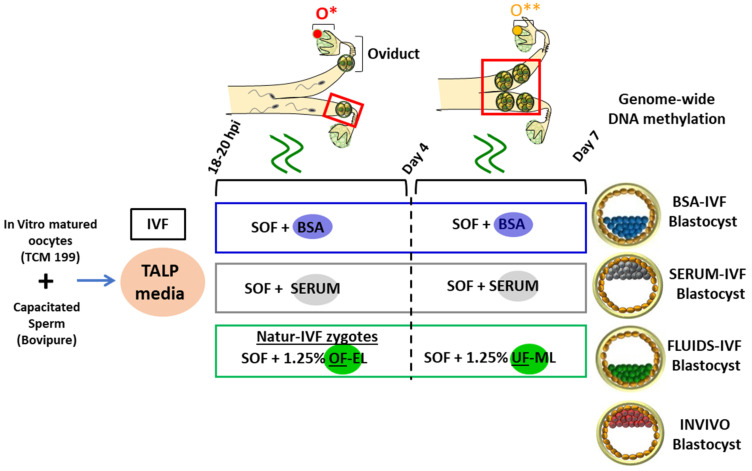
Schematic representation of the different steps of the IVF/EC system. Day 7 blastocysts in vitro were produced by oocyte in vitro maturation and fertilization, while presumptive zygotes were cultured from day 1 to 4 in SOF supplements either with 3 mg/mL BSA or 5% FCS or 1.25 OF, representing the time when the early embryo is in the oviduct and from day 4–7 in SOF supplements either with 3 mg/mL BSA or 5% FCS or 1.25 UF, representing the time when the embryo is in the uterine horn. Day 7 blastocysts in vivo were collected from superovulated donor heifers after flushing. O*: ovary with hemorrhagic corpus luteum; O**: early corpus luteum; OF-EL: oviductal fluid-early luteal; UF-ML: uterine fluid-mid luteal.

After 18–20 h post-insemination (hpi), presumptive zygotes were denuded of cumulus cells by vortexing for 3 min and then cultured in groups of 25 in 25 µL droplets of synthetic oviductal fluid (SOF) [36] with 4.2 mM sodium lactate, 0.73 mM sodium pyruvate, 30 µL/mL BME amino acids, 10 µL/mL minimum essential medium (MEM) amino acids and 1 mg/mL phenol red, covered with mineral oil at 38.5 °C and an atmosphere of 5% CO_2_, 5% O_2_ and 90% N_2_ with maximum humidity. Depending on the experiment (see Figure 10), the SOF was supplemented with BSA (3 mg/mL; BSA group) or reproductive fluids (1.25% *v/v* Natur ARTs OF-EL (Embryocloud, Murcia, Spain) from day 1 to 4 and 1.25% *v/v* NaturARTs UF-ML (Embryocloud, Murcia, Spain) from day 4 to 7 (FLUIDS group) or with 5% FCS (SERUM group). Cleavage rate and blastocyst yield were measured at 48 hpi and on day 7 (day 0 = day of IVF), respectively. Grade 1 blastocysts from all groups, based on the International Embryo Technology Society (IETS) embryo quality guidelines [37], were washed in PBS, and then the zona pellucida was removed by enzymatic digestion using pronase solution (5 mg/mL) for 2–3 min. Each blastocyst was washed 5 times in PBS, snap frozen in liquid nitrogen (NL2) and stored at −80 °C for further analysis.

### 3.2. In Vivo Collection of Embryos

Holstein cows (*n* = 3) received 2 mL (25 mg) intramuscular (i.m.) injection of prostaglandin F2 alpha (PGF2α; Estrumate, MSD Animal Health, S.L., Spain) (day 16) and after 7 days (day 9) an intravaginal P4 releasing device was inserted (CIDR-Zoetis, Madrid, Spain; 1.38 g P4). Two days later (day 7) cows received a 2 mL i.m. of a gonadotropin releasing hormone analog (GnRH; cystoreline, Ceva Sante Animale, equivalent to 0.1 mg gonadorelin). Super-stimulation treatments were initiated 3 days after GnRH injection (day 4) with a total dose of 360 mg NIH-FSH-P1 (follitropin; Vetoquinol Especialidades Veterinarias, S.A., Spain), divided into eight decreasing doses administered i.m. twice daily over 4 days (i.e., days 4 to 1). On day 2, cows received 3 mL of PGF (37.5 mg), and on day 1, CIDRs were removed. Twenty-four hours later cows received 2 mL i.m. of GnRH (day 0) and were inseminated 12 and 24 h later. On day 7, nonsurgical embryo collections and evaluations were performed. Grade 1 blastocyst were treated similar to the in vitro embryos for zona pellucida removal and stored at −80 °C for further analysis.

### 3.3. DNA Library Preparation Based on Post-Bisulfite Adapter Tagging

An adaptation of whole genome bisulfite sequencing that involves post-bisulfite adapter tagging (PBAT) [38] was used to analyze the methylome of individual cow blastocysts at single-base resolution on a genome-wide scale. Two blastocysts per group and sex were individually analyzed. Further modification of the method described in Smallwood et al. [39] was used to generate BS-seq libraries. Briefly, an individual blastocyst was lysed for 1 h in 1% sodium dodecyl sulfate (SDS) with proteinase K (100 μg/mL) and treated with bisulfite reagent using Imprint DNA modification kit (Sigma, MOD50). DNA was eluted in EB buffer, and one round of first strand synthesis was performed using a biotinylated oligo 1 (5-(Btn)CTACACGACGCTCTTCCGATC TNNNNNNNNN-3). Samples were further treated with exonuclease I, washed and eluted in 10 mM Tris-Cl and incubated with washed M-280 streptavidin dynabeads (Life Technologies, Carlsbad, CA, USA) to pull down the biotinilated fraction of DNA. Second strand synthesis was performed using oligo 2 (5′-TGC TGAACCGCTCTTCCGATCTNNNNNNNNN-3′), and samples were amplified for 15 PCR cycles using indexed iPCRTag reverse primer [39] with KAPA HiFiHotStart DNA polymerase (KAPA Biosystems, London, UK) and purified using 0.8× AgencourtAmpure XP beads (Beckman Coulter). Libraries were assessed for quality and quantity using high-sensitivity DNA chips on the Agilent Bioanalyzer, and the KAPA Library Quantification Kit for Illumina (KAPA Biosystems). The libraries generated from individual blastocysts for each experimental condition were prepared for 100 bp single-end sequencing on Illumina HiSeq 1000 and sequenced at three samples per lane.

### 3.4. Analysis of Methylation Data

Library sequence reads were mapped to the cow (genomic assembly UMD 3.1) genomes using the Bismark software (v.0.19; Babraham Institute). DNA methylation analysis was done using the SeqMonk software package (v.1.47.2; Babraham Institute; www.bioinformatics.babraham.ac.uk/projects/seqmonk/). For the unbiased analysis, tiles were defined in SeqMonk using sets of 100 merged consecutive probes from the probe set feature generator using CG_occurrences_UMD3.1.txt and duplicates removed over the feature. Then, the bisulphite quantitation pipeline was run over existing tiles, one minimum count to include position and 10 minimum observations to include feature and then combining using the mean transformed by matching distributions.

Then, for every pair-wise comparison, logistic regression statistic was applied (*p* < 0.05 after Benjamini and Hochberg correction with ratios recalculated from normalized quantitation), and finally results were filtered to require a consistent 10% change between two conditions.

For the targeted analysis, different genome feature annotations available from SeqMonk, and transposable elements information (LINE, SINE and LTR) from www.repeatmasker.org/species/bosTau.html (accessed date: 12 November 2020) were used. Promoter regions were defined as the region 1500 bp upstream of the transcription starting site (TSS) to 500 bp downstream of the TSS. Gene bodies were defined as the regions 500 bp downstream of the TSS to the polyadenylation site. First introns were extracted using subfeatures from Seqmonk.

### 3.5. Sexing of Embryos

Embryos were sexed by mid-throughput sequencing, based on the methylation reads observed at selected specific Y chromosome genes, such as ENSBTAG00000048172, UTY, DDX3Y, OFD1Y, EIF1AY and EIF2S3Y (Supplementary Figure S3). By using experimental groups containing exactly same proportion of males and females, we were able to identify the exact impact of the sex on the DNA methylation differences previously described. The first two females and two males per group were used for high-throughput sequencing.

### 3.6. Functional Analysis by PANTHER

Differentially methylated tiles were annotated by closest gene and then, using PANTER_v.14.0 statistical overrepresentation test (Fisher’s exact test and Bonferroni correction for multiple testing) was applied to obtain overrepresented GO biological functions and Panther Protein classes.

## 4. Discussion

DNA methylation is considered as an epigenetic biosensor or environmental footprint, which can reflect previous microenvironment exposures in a cell or tissue [40] and may be indicative of the embryo’s predisposition to certain diseases in adult life [1].

In this study, for the first time, whole-genome DNA methylation profiles on individual bovine blastocysts (in vivo- and in vitro-derived) were generated by a low-cell adaptation of the post-bisulphite adaptor-tagging (PBAT) method [38,41]. Novel data from this study complement the recent data reported for individual early embryos in this species [20,40]. However, more importantly, by adopting a novel approach to investigate the sex of the embryos, we uncover sex-specific methylation differences at blastocyst stage.

Differences in the global level of DNA methylation were found in single bovine blastocysts produced under different environmental conditions. Those embryos collected in vivo from the uterus of the donors showed the highest levels of methylation, very close to those produced in vitro with SERUM as protein supplement. By contrast, those embryos produced in vitro with either reproductive fluids or BSA as protein source showed almost 4% less global methylation. The interpretation of this result is complex, since serum and reproductive fluids are theoretically similar milieu of complex composition, while BSA is a single protein whose effect could not cover that of multiproteic solutions. Nonetheless, it should be noted that the concentration of the reproductive fluids in the culture media was lower (1.25%) than that of serum (5%), and this could explain the differences observed between these two groups of blastocysts. Until now, the technical protocol had not been developed that allows use of proportions of 5% of reproductive fluids in the culture media, and for this reason, we used 1.25%, the highest concentration without a toxic effect for the embryos according to our previous experience [21]. An increase in the proportion of reproductive fluids, once identifying the destabilizing factors altering their beneficial properties during the collection and storing processes, is a matter of future research and a potential tool to obtain embryos closest to their in vivo-derived counterparts.

The first observation derived from our results is that the lowest number of tiles with more than 10% methylation difference was found between the INVIVO and SERUM groups, coinciding with the most similar levels of global DNA methylation in all pairwise comparisons. By contrast, FLUIDS showed the highest proportion (among the three in vitro groups) of specifically DMT compared to INVIVO. Altogether, our data seem to point out that the FLUIDS blastocysts were, epigenetically speaking, the most distant from the INVIVO group. A quick look at the PCA plots (Figure 5), however, shows that SERUM blastocysts in both sexes were those clustering furthest from each other, suggesting a higher variability in this group of embryos. Such an observation corroborates the persistent claim of researchers about the inconsistence of the results when using serum as protein additive for the culture media, adducing batch to batch differences that make it an undesirable additive for the IVP laboratories [42]. The plausible influence of such variability in this group of blastocysts on the methylation analyses above discussed should be taken into account before extracting decisive conclusions.

The use of serum produces adverse effects at the transcriptional level [43,44,45], with a probable origin in aberrant DNA methylation of several imprinted genes [6,46,47,48], but nothing has been published about reproductive fluids because they have not been used until now. By contrast, the replacement of serum by BSA has not been proved to avoid most of the loss of imprinting problems associated with the ART [49], despite serum being used experimentally to increase the incidence of the associated overgrowth phenotype in in vitro-produced calves [6]. This controversy confirms the need to analyze the specific effects of each supplement (serum, BSA or reproductive fluids) at specific genomic regions.

To this end, we focused the analysis on CpGi in promoters and first introns, as they are well known transcriptional regulatory elements [32]. DMTs only showed an enrichment for first introns in SERUM and FLUIDS groups compared to the INVIVO group. This observation suggests that, in the context of global demethylation as happens in the early blastocyst, transcriptomic differences between INVIVO and in vitro groups are more influenced by differential methylation in the first intron than in CpGi in promoters. Previous reports have shown a lack of correlation between DNA methylation at the promoter region and gene expression during demethylation of PGCs or early embryos [20,50]. Thus, it should be noted that this result is important for future analysis about the impact of the culture conditions and the correlation of DNA methylation and gene expression in this context.

Repetitive elements can regulate transcription, but even more importantly, they are sensible to ART, with LINE1 showing more sensitivity than other regions in mouse and human [51,52]. During in vitro culture, the cow genome undergoes drastic demethylation from gametes to the early blastocyst stage, and LINEs, SINEs and LTRs follow a similar trend [40]. As putative indicators of the in vitro-derived stress, we analyzed methylation values in LINEs and LTRs from in INVIVO versus in vitro-derived blastocysts. Curiously, the SERUM group turned out to be the most affected, as DMTs showed an enrichment for LINEs and LTRs. How this increased aberrant methylation at the transposons in the presence of serum can affect further phenotypical features during in utero development, and after birth remains as a future objective to be investigated.

Indeed, global protein composition of the culture media has been shown to play a role in the regulation of epigenetic marks during in vitro embryo culture, as demonstrated by the relationship between childbirth weight and the protein source in embryo culture media [53], or the increase of implantation rate and births by protein enrichment of the media [54]. However, none of these studies has considered the possibility that the sex of the embryo can influence the differences. Despite the undeniable effect of the in vitro culture, and of the culture media composition, on the DNA methylation pattern of the bovine blastocysts, it was evident that sex determination produced a stronger bias in the results (Figure 5B,C,E), thus deserving an additional analysis to investigate this effect.

Even though the notion that there are sex-determining factors upstream of gonadogenesis has been restated recently (reviewed by [55]), it is known for a long time that there are dissimilarities between male and female preimplantation embryos in gene expression [23,45], responses to cytokines [56] or susceptibility to environmental factors or stress [57,58].

In this sense, female embryos have been proposed to be more resistant to suboptimal in vitro conditions than males [52,58] and, particularly in bovine blastocysts, differences in glucose metabolism and in the kinetics of development between both sexes are striking [33], but none of these observations have been linked, until now, to a different reprogramming of epigenetic marks during the preimplantation development.

Sexual dimorphism in DNA methylation levels is a recurrent epigenetic feature in different cell types, including embryonic stem cells [59,60]. However, contrary to the extensive study of transcriptional sexual dimorphism in embryos, sexually-dimorphic DNA methylation patterns is almost unexplored in preimplantation mammalian embryos. Sex differences in degree of methylation at specific loci in bovine blastocyst [33], in 5-methylcitosine immunolabeling at the 8-cell stage [61] or differential DNA methylation in male and female blastocysts after CSF2 supplementation in in vitro culture [18] suggest sexual dimorphism in DNA methylation by the blastocysts stage. In the current study, male and female blastocysts clustered separately for each group but in the vicinity of their counterparts, with INVIVO blastocysts showing less distance between sexes. Curiously, two of the female embryos in the FLUIDS group and one in the SERUM group were far from the rest in the PCA. This fact could reflect a higher development programming of the female embryos under in vitro conditions and would explain the higher resistance of the female embryos to suboptimal in vitro conditions than males [58]. Additionally, human male embryos appear more vulnerable than female ones to in vitro condition-related global changes in DNA methylation, even in opposite sex twins [52].

Further consequences of higher development programming remain totally unknown but could be linked with the sex bias reported in some diseases with early developmental origins, such as congenital heart defects, blood pressure and insulin resistance, or the consequences of the maternal undernutrition associated with sex-specific alterations in fetal heart development [62,63,64].

Several studies have reported that molecular and phenotypic outcomes of adverse in utero conditions are often more prominent in male than female offspring [65]. Although the basis for this observation has not been uncovered in most studies, the presence of two X chromosomes in females could confer some health advantages. How the reported differences in the developmental programming between male and female could affect this process requires further studies.

The global methylation values reported in the present study are close to those reported by our group for non-sexed bovine in vitro-derived blastocysts [40] and higher than data previously reported in bovine non-sexed blastocysts using reduced representation bisulfite sequencing (RRBS) [66]. Here, it is important to note that RRBS is biased towards the CG-rich and hence the less methylated part of the genome.

Results also displayed higher methylation in INVIVO female blastocysts than male blastocysts, which could be explained by the inactivation, at least partially, through DNA methylation of the second copy of X chromosome at this time [23]. Nonetheless, higher 5-methylcitosine immunolabeling in in vitro-derived male blastocysts has also been reported, which agrees with our data for the BSA and FLUIDS groups [61]. As has been widely reported for embryonic stem cells, the epigenetic state of the X-chromosome varies between cell lines, but even cell passages of a cell line and culture conditions affect this state of the X-chromosome [67,68,69,70]. This observation supports the described disparities in the methylation level between male and female blastocysts using different culture conditions and versus INVIVO-derived embryos and points to the need to find standardized conditions for all the culture media used in assisted reproduction for the sake of higher consistency of results.

In the unbiased analysis, the number of tiles differentially methylated between male and female blastocysts (27205–38087) was in the same order of magnitude as data previously reported (31706 DMRs) using a bovine methylome array [18]. Between these SDMTs, gene ontology analysis revealed the presence of critical factors for reproduction, developmental process and cell proliferation. Additionally, several genes with roles in DNA methylation (DNMT1, UHRF2, TET1, TET2, TET3 and ZFP57) were included in this set for INVIVO blastocysts, which suggests that sex-specific differences in the INVIVO blastocysts are supported, at least partially, by epigenetic regulation of the DNA methylation machinery and developmental process.

Considering that differentially expressed genes in male and female bovine blastocysts show a clear chromosomal location bias towards the X chromosome [23,45], we analyzed chromosomal distribution of the SDMTs. More than 10% of the SDMTs (2652 over 25,717 SDMTs) in the INVIVO group are located in the X chromosome, which represents more than double the number of DMTs located in any other chromosome. The X-chromosome bias of the SDMT and identification of factors related with DNA methylation machinery, reproduction, meiotic cell cycle and gamete generation strengthens the link between sexual transcriptomic and epigenetic dimorphism in blastocysts. Moreover, other epigenetic marks such as histone modifications have revealed epigenetic dimorphism recently, as it can be modified in the trophoctoderm by embryo sex [71]. Furthermore, there are other organisms in nature, as aphids, which in the absence of confounding genetic variation, have revealed that methylation regulates phenotypic plasticity and is intrinsically linked to sexual dimorphism [72]. Nonetheless, the genuine force driving these sex-specific dissimilarities in blastocyst is still unrevealed in mammals.

In conclusion, our results show that bovine preimplantation blastocysts obtained in vivo showed the highest global levels of methylation, close to those produced in vitro with serum. However, the serum group was the most affected during the repetitive element analysis. On the other hand, sex determination produced a stronger bias in DNA methylation than embryo origin, with in vivo blastocysts showing a lesser distance between male and female blastocysts. Contrarily, higher distances were observed in some in vitro-derived female embryos, which could be linked with higher programming and higher resistance of the female embryos to suboptimal in vitro conditions. In fact, sexually dimorphic methylated tiles include critical factors for reproduction, the developmental process, cell proliferation and DNA methylation machinery, which could be involved in this differential programming.

## Figures and Tables

**Table 1:** Differentially methylated regions (tiles 100 CpGs) by logistic regression (*p* < 0.05) in each pair-wise comparison. From these tiles, DMTs with more than 10% methylation values differences were filtered.

DMTs (100 CpG Tiles)	# Tiles (% over Total Titles) Differentially Methylated, *p* < 0.05	# Tiles Differentially Methylated with > 10% Methylation dif (% over DMTs)		
INVIVO vs. BSA	68,386 (25.13%)	15,930 (23.29%)	Up in INVIVO	7,396
			Up in BSA	8,534
INVIVO vs. SERUM	70,908 (26.06%)	13,950 (19.67%)	Up in INVIVO	7,199
			Up in SERUM	6,751
INVIVO vs. FLUIDS	88,359 (32.48%)	20,588 (23.30%)	Up in INVIVO	9,796
			Up in FLUIDS	10,792
FLUIDS vs. BSA	49,775 (18.30%)	9,871 (19.83%)	Up in FLUIDS	5,893
			Up in BSA	3,978
FLUIDS vs. SERUM	57,866 (21.27%)	9,333 (16.13%)	Up in FLUIDS	5,364
			Up in SERUM	3,969
BSA vs. SERUM	57,212 (21.03 %)	8,652 (15.13 %)	Up in BSA	4,235
			Up in SERUM	4,417

**Table 2:** Sexually dimorphic methylation tiles (*p* < 0.05 after correction and methylation values differences over 10%) overlapping genes involved in the DNA methylation machinery.

	INVIVO	SERUM	FLUIDS	BSA
DNMT1	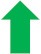 females		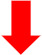 females	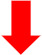 females
DNMT3A			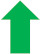 females	
DNMT3B				
ZFP57	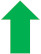 females			
TET1	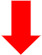 females			
TET2	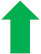 females	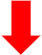 females	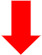 females	
TET3	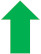 females	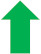 females		
UHRF2	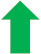 females		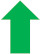 females	

**Table 3:** DMTs were calculated by logistic regression (*p* < 0.05) for each pairwise comparison. From these DMTs, tiles with methylation differences over 10% were selected and split in tiles with increased methylation for each group.

DMTs (Total 272053)	FEMALES							MALES						
	*p* < 0.05	+ Methylation Difference > 10%					%	*p* < 0.05	+ Methylation Difference > 10%					%
				% Over Total							% Over Total			
INVIVO vs. FLUIDS	78,979	47,969	*	17.63	Up in INVIVO	22,148	46.17	65,570	30,849	*	11.34	Up in INVIVO	15,122	49.02
					Up in FLUIDS	25,821	53.83					Up in FLUIDS	15,727	50.98
INVIVO vs. BSA	56,015	30,571		11.24	Up in INVIVO	15,776	51.60	75,793	40,885		15.03	Up in INVIVO	20,864	51.03
					Up in BSA	14,795	48.40					Up in BSA	20,021	48.97
INVIVO vs. SERUM	35,762	22,185	*	8.15	Up in INVIVO	12,999	58.59	77,265	33,389	*	12.27	Up in INVIVO	16,981	50.86
					Up in SERUM	9,186	41.41					Up in SERUM	16,408	49.14
FLUIDS vs. BSA	71,191	26,075	*	9.58	Up in FLUIDS	21,218	81.37	52,545	15,510	*	5.70	Up in FLUIDS	12,350	79.63
					Up in BSA	4,857	18.63					Up in BSA	3,160	20.37
FLUIDS vs. SERUM	49,662	27,937	*	10.27	Up in FLUIDS	13,010	46.57	67,148	27,412	*	10.08	Up in FLUIDS	14,487	52.85
					Up in SERUM	14,927	53.43					Up in SERUM	12,925	47.15
SERUM vs. BSA	48,251	26,563	*	9.76	Up in SERUM	12,434	46.81	71,791	32,638	*	12.00	Up in SERUM	15,368	47.09
					Up in BSA	14,129	53.19					Up in BSA	17,270	52.91

Significant differences in the number of DMTs with methylation difference >10% between each two groups were calculated by Chi-square (* *p* < 0.001) with respect to the DMTs between INVIVO and BSA groups, which was considered as the control for the in vitro groups.

## Data Availability

The data presented in this study are available on request from the corresponding author.

## Supplementary Materials

The following are available online at https://www.mdpi.com/article/10.3390/ijms22126426/s1.
