# Supplementary material for: Culture Medium and Sex Drive Epigenetic Reprogramming in Preimplantation Bovine Embryos
Source: Int J Mol Sci. 2021 Jun 15;22(12):6426. doi: 10.3390/ijms22126426 (PMC8232708; doi:10.3390/ijms22126426)
Supplement: Supplementary file 1 [file ijms-22-06426-s001.zip › Supplementary Figure S1.pdf]

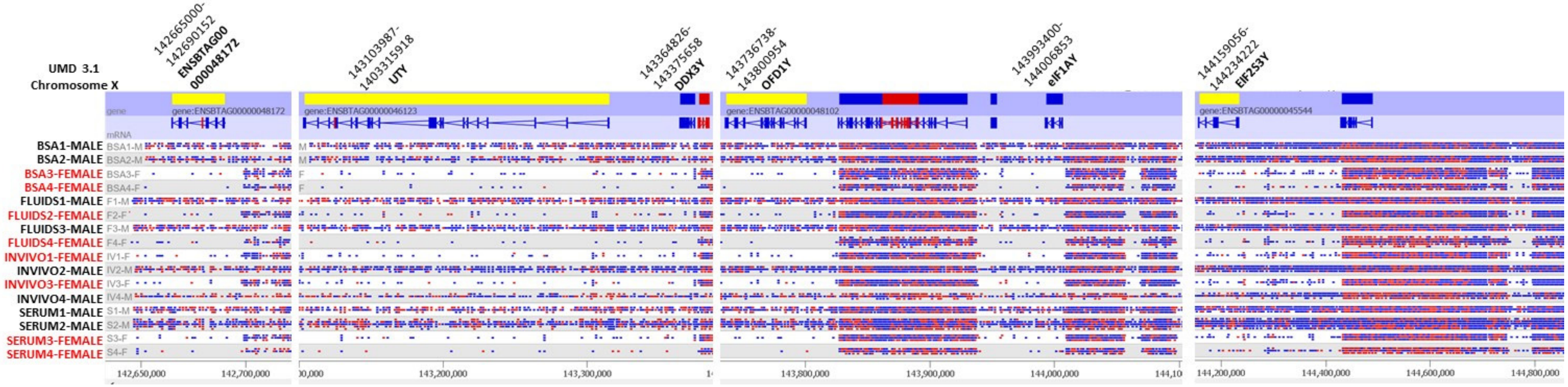

Supplementary Figure S1. Mid-throughput sequencing. Methylation reads observed at selected specific Y chromosome genes ENSBTAG00000048172, UTY, DDX3Y, OFD1Y, EIF1AY and EIF2S3Y.
