# Supplementary material for: Culture Medium and Sex Drive Epigenetic Reprogramming in Preimplantation Bovine Embryos
Source: Int J Mol Sci. 2021 Jun 15;22(12):6426. doi: 10.3390/ijms22126426 (PMC8232708; doi:10.3390/ijms22126426)
Supplement: Supplementary file 1 [file ijms-22-06426-s001.zip › Supplementary Figure S3.pdf]

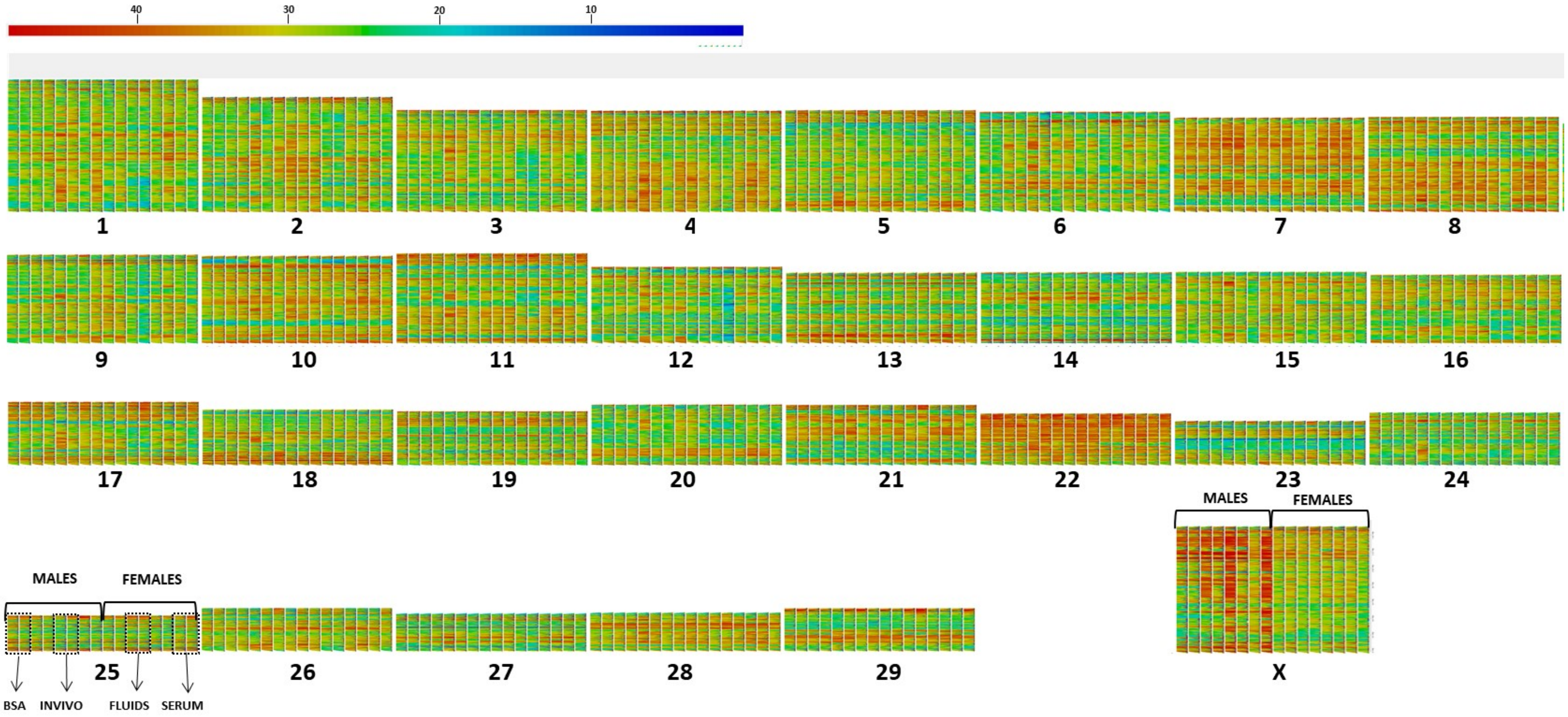

Supplementary Figure S3. Domainogram shows global view of the methylation (%) by chromosome. Two samples per groups and sex are shown in the following order: BSA, INVIVO, FLUIDS, SERUM. Numbers under pictograms label the chromosome.
