# Supplementary material for: Culture Medium and Sex Drive Epigenetic Reprogramming in Preimplantation Bovine Embryos
Source: Int J Mol Sci. 2021 Jun 15;22(12):6426. doi: 10.3390/ijms22126426 (PMC8232708; doi:10.3390/ijms22126426)
Supplement: Supplementary file 1 [file ijms-22-06426-s001.zip › Supplementary Table S1.pdf]

Supplementary Table S1. Number of unique alignments and the coverage of CpGs (≥1 read). Total number of CpG in cow genome: 27203575

Sample	Number of unique alignments	Coverage of CpGs (≥1 read).
IV1	20171279,00	0,74149368
IV2	20572520,00	0,75624325
IV3	20334161,00	0,7474812
IV4	13178335,00	0,48443394
1BSA	20659761,00	0,75945022
2BSA	19809100,00	0,72818003
3BSA	19634340,00	0,72175587
4BSA	13944730,00	0,51260652
1F	21427275,00	0,78766394
2F	20172039,00	0,74152162
3F	20357055,00	0,74832278
4F	15656814,00	0,57554252
1S	21152156,00	0,7775506
2S	22294697,00	0,81955026
3S	19923342,00	0,73237955
4S	12966981,00	0,47666459
