# Supplementary material for: Culture Medium and Sex Drive Epigenetic Reprogramming in Preimplantation Bovine Embryos
Source: Int J Mol Sci. 2021 Jun 15;22(12):6426. doi: 10.3390/ijms22126426 (PMC8232708; doi:10.3390/ijms22126426)
Supplement: Supplementary file 1 [file ijms-22-06426-s001.zip › Supplementary Table S2.pdf]

Supplementary Table S2. Sequencing output for DNA libraries from bovine blastocysts.

SEX	ID	Total reads	Alignments analysed	Unique alignments	Total C's analysed	Methylated C's in CpG context	% Methyl CpG context	% Methyl CHG context	% Methyl CHH context
M	S1	144.301.680	86.655.478	59.322.846	1.183.174.466	23.308.282	30,1	0,5	0,4
M	S2	178.363.967	102.130.846	72.901.316	1.448.721.372	19.924.406	21,1	0,6	0,5
F	S3	111.238.518	62.706.076	45.237.485	895.246.548	16.009.000	27,5	0,6	0,5
F	S4	34.605.670	19.935.226	17.142.606	342.258.539	7.447.400	33,6	0,8	0,8
M	F1	140.982.868	80.105.559	59.379.806	1.185.238.772	21.800.801	27,9	0,6	0,5
F	F2	124.769.934	71.328.446	49.025.028	975.673.724	16.563.674	25,6	0,6	0,5
M	F3	120.824.818	70.126.407	50.258.855	996.551.285	17.155.334	25,8	0,7	0,6
F	F4	50.734.099	28.936.845	24.801.404	384.439.364	6.177.895	20,5	1,3	1,1
F	IV1	133.089.200	78.112.310	49.843.292	986.094.949	19.763.503	30,9	0,7	0,6
M	IV2	133.953.929	76.193.149	52.478.860	1.038.220.592	16.136.842	23,6	0,6	0,6
F	IV3	137.364.964	79.291.415	50.343.204	995.860.670	19.309.470	29,5	0,6	0,6
M	IV4	39.889.995	25.015.670	20.308.532	391.377.389	7.480.860	34	0,9	0,8
M	BSA1	133.179.441	78.091.477	52.593.450	1.048.510.242	18.295.603	26,9	0,6	0,5
M	BSA2	113.715.292	66.115.072	46.088.061	921.011.398	17.018.131	27,7	0,6	0,5
F	BSA3	131.733.229	75.433.916	44.662.517	887.731.668	11.248.651	19,5	0,6	0,5
F	BSA4	38.571.634	22.463.701	19.505.630	384.439.364	6.720.012	27,5	1,7	1,5
