# Supplementary figures and images for: Culture Medium and Sex Drive Epigenetic Reprogramming in Preimplantation Bovine Embryos
Source: Int J Mol Sci. 2021 Jun 15;22(12):6426. doi: 10.3390/ijms22126426 (PMC8232708; doi:10.3390/ijms22126426)

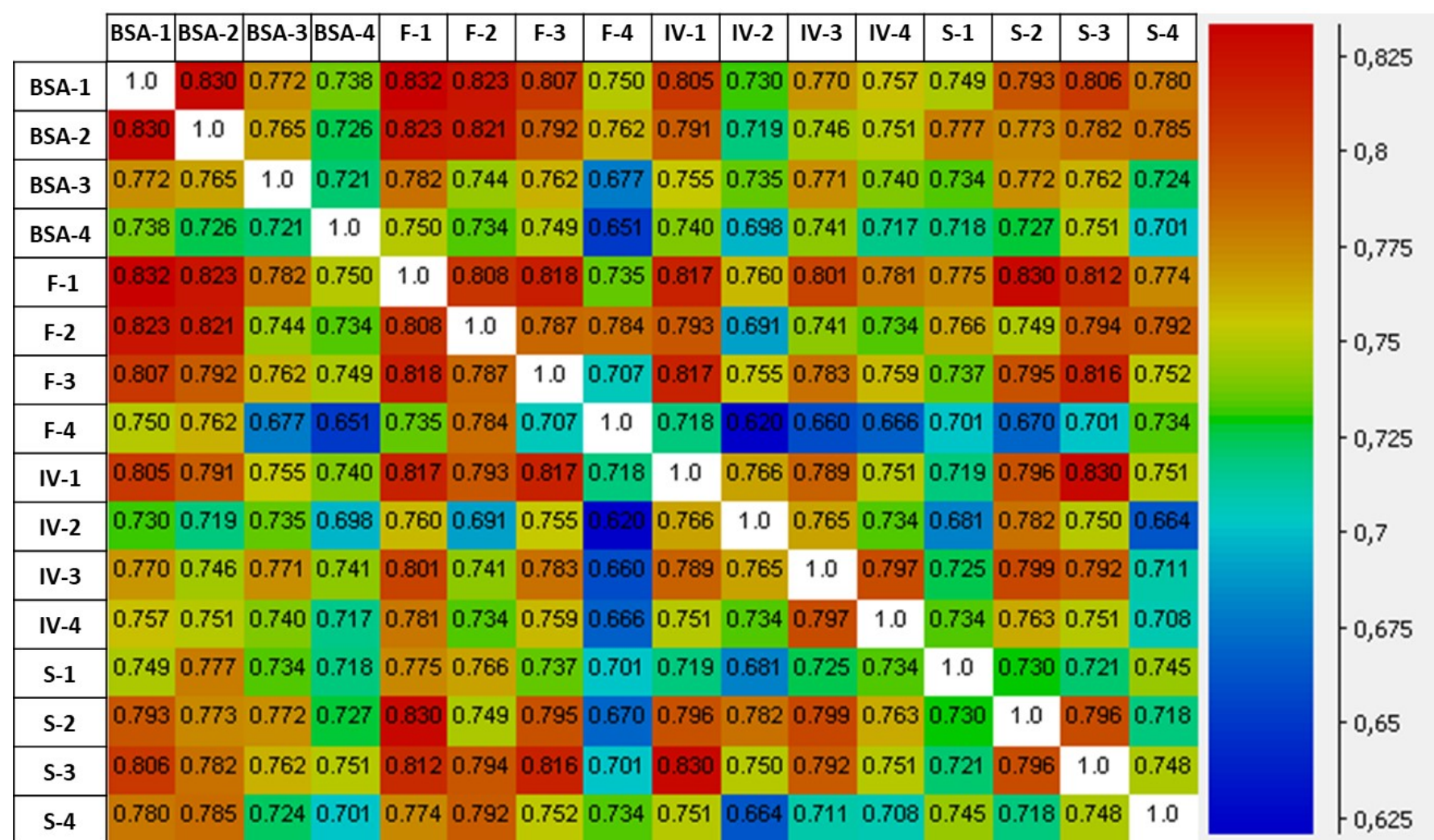

**Supplementary Figure S2.** Correlation matrix for all individual samples.

Supplement: Supplementary file 1 [file ijms-22-06426-s001.zip › Supplementary Figure S2.pdf]
